# Associations of meeting 24-h movement behavior guidelines with cognitive difficulty and social relationships in children and adolescents with attention deficit/hyperactive disorder

**DOI:** 10.1186/s13034-023-00588-w

**Published:** 2023-03-27

**Authors:** Alyx Taylor, Chuidan Kong, Zhihao Zhang, Fabian Herold, Sebastian Ludyga, Sean Healy, Markus Gerber, Boris Cheval, Matthew Pontifex, Arthur F. Kramer, Sitong Chen, Yanjie Zhang, Notger G. Müller, Mark S. Tremblay, Liye Zou

**Affiliations:** 1 School of Rehabilitation, Sport and Psychology, AECC University College, Bournemouth, BH5 2DF UK; 2 Body-Brain-Mind Laboratory; The Shenzhen Humanities & Social Sciences Key Research Bases of the Center for Mental Health School of Psychology, Shenzhen University, Shenzhen, 518061 China; 3 Research Group Degenerative and Chronic Diseases, Movement, Faculty of Health Sciences Brandenburg, University of Potsdam, 14476 Potsdam, Germany; 4 Department of Sport, Exercise, and Health, University of Basel, 4052 Basel, Switzerland; 5 Community Health Academic Group, School of Nursing, Psychotherapy and Community Health, Dublin City University, Dublin, 9 Ireland; 6 Swiss Center for Affective Sciences, University of Geneva, Geneva, Switzerland; 7 Departments of Kinesiology, Michigan State University, East Lansing, USA; 8 Center for Cognitive and Brain Health, Northeastern University, Boston, MA 02115 USA; 9 Beckman Institute, University of Illinois at Urbana-Champaign, Champaign, IL 61820 USA; 10 Institute for Health and Sport, Victoria University, Melbourne, 8001 Australia; 11 Physical Education Unit, School of Humanities and Social Science, The Chinese University of Hong Kong, Shenzhen, 518172 China; 12 Healthy Active Living and Obesity Research Group, CHEO Research Institute, Ottawa, ON K1H 8L1 Canada; 13 Department of Pediatrics, University of Ottawa, Ottawa, ON K1N 6N5 Canada

## Abstract

**Background:**

Evidence-based 24-h movement behavior (24-HMB) guidelines have been developed to integrate recommendations for the time spent on physical activity, sedentary behavior, and sleep. For children and adolescents, these 24-HMB guidelines recommend a maximum of two hours of recreational screen time (as part of sedentary behavior), a minimum of 60 min per day of moderate to vigorous physical activity (MVPA), and an age-appropriate sleep duration (9–11 h for 5 to 13-year-olds; 8–10 h for 14 to 17-year-olds). Although adherence to the guidelines has been associated with positive health outcomes, the effects of adhering to the 24-HMB recommendations have not been fully examined in children and adolescents with attention eficit/hyperactive disorder (ADHD). Therefore, this study examined potential associations between meeting the 24-HMB guidelines and indicators of cognitive and social difficulties in children and adolescents with ADHD.

**Methods:**

Cross-sectional data on 3470 children and adolescents with ADHD aged between 6 and 17 years was extracted from the National Survey for Children’s Health (NSCH 2020). Adherence to 24-HMB guidelines comprised screen time, physical activity, and sleep. ADHD-related outcomes included four indicators; one relating to cognitive difficulties (i.e., serious difficulties in concentrating, remembering, or making decisions) and three indicators of social difficulties (i.e., difficulties in making or keeping friends, bullying others, being bullied). Logistic regression was performed to determine the associations between adherence to 24-HMB guidelines and the cognitive and social outcomes described above, while adjusting for confounders.

**Results:**

In total, 44.8% of participants met at least one movement behavior guideline, while only 5.7% met all three. Adjusted logistic regressions further showed that meeting all three guidelines was associated with lower odds of cognitive difficulties in relation to none of the guidelines, but the strongest model included only screen time and physical activity as predictors (OR = 0.26, 95% CI 0.12–0.53, *p* < .001). For social relationships, meeting all three guidelines was associated with lower odds of difficulty keeping friends (OR = 0.46, 95% CI 0.21–0.97, *p* = .04) in relation to none of the guidelines. Meeting the guideline for screen time was associated with lower odds of being bullied (OR = 0.61, 95% CI 0.39–0.97, *p* = .04) in relation to none of the guidelines. While screen time only, sleep only and the combination of both were associated with lower odds of bullying others, sleep alone was the strongest predictor (OR = 0.44, 95% CI 0.26–0.76, *p* = .003) in relation to none of the guidelines.

**Conclusion:**

Meeting 24-HMB guidelines was associated with reduced likelihood of cognitive and social difficulties in children and adolescents with ADHD. These findings highlight the importance of adhering to healthy lifestyle behaviors as outlined in the 24-HMB recommendations with regard to cognitive and social difficulties in children and adolescents with ADHD. These results need to be confirmed by longitudinal and interventional studies with a large sample size.

## Highlights

1. Meeting the combination of all three 24-HMB guidelines, or a combination of screen time and physical activity, was associated with reduced odds of serious difficulties in concentrating, remembering, or making decisions.
2. Meeting the combination of all three 24-HMB guidelines was associated with reduced odds of difficulties making and keeping friends.
3. Meeting the 24-HMB guideline for screen time was associated with reduced odds of being bullied.
4. Meeting the individual guidelines for screen time or sleep duration or a combination of both was associated with reduced odds of bullying others.

## Introduction

Attention deficit/hyperactive disorder [1] is a common neurodevelopmental disorder that affects both children and adults and is characterized by deficits in the domains of attention and hyperactivity- impulsivity [1–4]. Approximately 5 to 7% of children and adolescents are diagnosed with ADHD worldwide [5, 6] and an additional 5% who exhibit symptoms that do not reach diagnostic level [7]. The symptoms of ADHD can have a range of negative consequences. In children and adolescents, the symptoms can reduce the quality of their social interactions, academic and learning activities [8], hindering cognitive development [9], and educational achievement [10, 11]. In addition, there is an increased risk of other mental and physical health conditions including anxiety [12], depression [13], and obesity [14]. When symptoms of ADHD persist into adulthood, difficulties with social life, employment [15], or law enforcement [16] can emerge.

Pharmacological treatments, primarily stimulant medication [17], behavioral therapy, and parent training are standard interventions for ADHD [18]. However, approximately 25% of ADHD patients do not respond to stimulant medication and some are unable to tolerate the side effects [19]. Likewise, for some individuals with ADHD, the behavioral interventions are sufficient to manage their symptoms, while others do not seem to benefit in the same way [1, 2, 20]. Therefore, other non-pharmacological interventions have been investigated, including cognitive training (e.g., working memory training) [21, 22] and regular physical activity [23].

There is mounting evidence that physical activity is beneficial for behavior and cognitive performance of children and adolescents with ADHD [24, 25, 48]. In addition to physical activity, other lifestyle factors such as sleep and sedentary behavior may also be related to the symptomatology associated with ADHD [26–29]. For example, in the general population, lower levels of sedentary behaviors, mainly recreational screen time [30], and optimal sleep duration [31] have been positively and independently related to academic achievement in children and adolescents [32]. There is also evidence that these three movement behaviors (i.e., physical activity, sedentary behavior, and sleep) are codependent and thus should be examined simultaneously [33–35]. Together the above-mentioned evidence indicates that integrating non-pharmacological or lifestyle-related interventions aiming to positively influence sedentary behavior, sleep duration and the level of regular physical activity may benefit children and adolescents with ADHD.

The 24-hour movement behavior (24-HMB) guidelines for children and adolescents may serve as the basis for considering multi-behavioral interventions that integrate physical activity, sedentary behavior, and sleep [36–42]. Specifically, the guidelines recommend a limit of two hours of non-educational screen time, and a minimum of sixty minutes of moderate-to-vigorous physical activity (MVPA) daily, as well as age-appropriate sleep duration each night. Previous research has shown that children and adolescents with ADHD are typically less active than their peers in the general population [43–45]. For example, Friel et al. (2020) reported, based on national survey on children and adolescents aged 6–17 years in the US, that the majority (91.2%) met at least one of the 24-HMB guidelines, but only 8.8% met all three 24-HMB recommendations [46]. In comparison, Wang et al. (2022) using the data of the 2018-19 NSCH (National Survey of Children's Health dataset) survey, observed that less than half (46.8%) of the children and adolescents with ADHD (aged between 6 and 17 years) met at least one guideline and that 6.5% met all three 24-HMB recommendations [47]. In summary, children and adolescents with ADHD are less likely to adhere to the 24-HMB recommendations than their peers without ADHD.

While positive associations of meeting the 24-HMB guidelines in relation to social interaction and cognitive function have been found in the general population [49–51], these have not been examined specifically in children and adolescents with ADHD. Furthermore, as academic performance is closely related to social interaction and cognitive function, it could be particularly helpful for the young people with ADHD to adhere to the 24-HMB guidelines. Based on the current the literature, we hypothesized that adhering to all three components of the 24-HMB guidelines would be associated with a reduced likelihood of cognitive and social difficulties in children and adolescents with ADHD, when controlling for demographic, socioeconomic and other medical factors. Therefore, this study examined the associations between meeting the 24-HMB guidelines for physical activity, sleep duration and screen time, and measures of cognitive and social difficulties in children and adolescents with ADHD. Results of this study will help health professionals and school administrators deepen an understanding about symptomatic management of children and adolescents with ADHD.

## Method

### Study design and data source

In this cross-sectional study, we used data from the United States’ 2020 NSCH survey that was collected from June 2020 to January 2021. The survey provides data on health-related measures of the children, their families, and communities, the prevalence of disease type, and associated healthcare needs. In the 2020 NSCH survey, approximately 240,000 households from 50 states and the District of Columbia were invited to complete the screening questionnaire, which selects households with children. Respondents, who are primary caregivers of the child in the household, were invited to participate. Of those who responded, 51,107 were eligible to continue to the data collection and 42,777 provided complete responses. The ethical approval and process for obtaining consent to participate are summarized below under the subheading Declaration, and given in full detail in the 2020 NSCH methodology report [52].

### Participants and procedure

For the current study, data were retrieved on children with a diagnosis of ADHD between the ages of 6 and 17 years and their families from the NSCH 2020 database. The inclusion criteria for the current study were positive responses by the parent or guardian of the child to the following survey items: (i) “Has a doctor or other health care provider ever told you that this child has attention deficit/hyperactivity disorder, that is, ADD or ADHD?”; (ii) “If yes, does the child currently have the condition?” These criteria were used to select 3740 children with ADHD and their families from the database. The survey requires the respondents to report on only one child, even when more than one child has the same diagnosis. The demographic data and variables for the exposure of interest and outcomes were then selected for further analysis.

### Demographic and medical data

The demographic data selected included the child’s age, sex, ethnicity, preterm birth status, overweight status, ADHD-related medication and behavioral treatment, household poverty level, and the highest level of education of primary caregivers (Table 1). According to the US federal poverty level, family income was coded to one of two levels. The variable called overweight was collected by asking the caregivers to report whether or not the child had been identified by their doctor or another healthcare worker as being overweight.

**Table 1:** Participant characteristics

ADHD (*n* = 3470)	
Characteristics	Value^1^
Age (M, SD)	11.97 (3.48)
Sex (n, %)	
Male	2376 (69.55)
Female	1094 (30.45)
Ethnicity (n, %)	
White	2839 (69.18)
Black or African American	250 (17.29)
American Indian/Alaska native	26 (0.59)
Asian	55 (1.07)
Native Hawaiian & other pacific islander	13 (0.86)
Two or more ethnic groups	287 (11.01)
Born 3 weeks or more before due dates (n, %)	
Yes	511 (14.47)
No	2959 (85.53)
Overweight status (n, %)	
Yes	469 (14.95)
No	3001 (85.05)
ADHD severity (n, %)	
Mild	1527 (43.20)
Moderate	1566 (42.29)
Severe	377 (14.52)
ADHD related medication or treatment (n, %)	
Behavioural treatment only	493 (14.39)
Medication only	1008 (26.18)
Behavioural treatment and medication	1047 (33.15)
Neither	905 (26.27)
Household poverty level (n, %)	
≤ 0–99% federal poverty level	457 (18.67)
≥ 100% federal poverty level	3013 (81.33)
Highest education level among reported adults (n, %)	
Less than high school	86 (7.02)
High school	540 (23.74)
Some college or associated degree	879 (24.06)
College degree or higher	1965 (45.18)
Adherence to the 24-h movement guidelines (n, %)	
None	841 (27.56)
Screen time	314 (10.87)
Sleep	1118 (26.87)
Physical activity	124 (4.93)
Screen time + Sleep	574 (14.69)
Screen time + Physical activity	116 (4.00)
Sleep + Physical activity	182 (5.40)
All	201 (5.68)
Serious difficulty concentrating, remembering, or making decisions (n, %)	
Yes	1858 (55.57)
No	1612 (44.43)
Difficulty making or keeping friends (n, %)	
No difficulty	1545 (45.96)
A little difficulty	1322 (36.54)
A lot of difficulty	603 (17.49)
Bully others (n, %)	
Never	2374 (69.21)
1 to 2 times in the past 1 year	762 (19.73)
1 to 2 times per month	198 (5.16)
1 to 2 times per week	78 (2.73)
Almost every day	58 (3.17)
Being bullied (n, %)	
Never	1363 (40.85)
1 to 2 times in the past 1 year	1190 (33.67)
1 to 2 times per month	452 (11.15)
1 to 2 times per week	270 (7.40)
Almost every day	195 (6.93)

Values are mean (SD) or n (%). N represents unweighted sample counts and % is weighted to the US population

### Exposure of interest: meeting the 24-HMB guidelines

The exposure of interest was meeting the 24-HMB guidelines [41]. These consist of three recommendations: Sufficient sleep for the age group (9–11 h for 5- to 13-year-olds; 8-10 hours for 14- to 17-year-olds); a minimum of 60 min per day of MVPA; and no more than 2 h per day of recreational screen-time. For MVPA, the survey question was: “During the past week, on how many days did this child exercise, play a sport, or participate in physical activity for at least 60 min?” In the current study, the answer of 7 days was considered as meeting the 24-HMB guidelines while lower levels (i.e., 6 or less days) was considered as not meeting the 24-HMB recommendations concerning physical activity. The question related to sleep was: “During the past week, how many hours of sleep did this child get on most weeknights?” With regard to each age range, the minimum number of hours was used to gauge whether the children/adolescents met the sleep recommendation or not. Screen-time was evaluated using the question: “On most weekdays, about how much time did this child spend in front of a TV, computer, cell phone, or other electronic device watching programs, playing games, accessing the internet, or using social media? (Do not include time spent doing schoolwork.)”. Responses of 2 h or less were considered as meeting the screen time recommendation, while any other responses were classified as not meeting the 24-HMB guidelines.

In this study, the number of guidelines that each child met (0 to 3), was used as a continuous variable for further statistical analyses. In addition, four combinations of whether the 24-HMB guidelines were met or not were used as separate variables in follow-up analyses: each pair (physical activity and screen-time; physical activity and sleep; screen-time and sleep) and meeting all guidelines.

### Outcomes: cognitive and social difficulties

Cognitive difficulties were evaluated by the question “Does the child have serious difficulty concentrating, remembering, or making decisions?” The binary response options were *yes* or *no*. In addition, social difficulties were evaluated using three questions: (i) “Does the child have difficulty in making friends or keeping friends?” The response had three levels, with the options: *no, a little,* or *a lot*. (ii) “During the past 12 months, how often did this child bully others, pick on them, or exclude them?” (iii) “During the past 12 months, how often was this child bullied, picked on, or excluded by other children?” For the second and third questions, the response options had five levels: *never, 1 or 2 times per year, 1 or 2 times per month, 1 or 2 times per week,* and *almost every day*.

### Confounders

The potential confounders included in the statistical analyses were age, sex, ethnicity whether or not the child had been born 3 or more weeks earlier than their due date, severity level of ADHD symptoms (mild, moderate, and severe), and ADHD-related medication and/or treatment (neither, behavioral treatment only, medication only, behavioral treatment and medication).

### Statistical analysis

Descriptive statistics were calculated for all variables. Continuous variables were described with means and standard deviations, and categorical variables were described using unweighted sample counts and weighted percentages. Multiple logistic regression was used to estimate the odds ratios (with 95% confidence intervals) between meeting 24-HMB guidelines and its component recommendations and four outcomes, including one indicator of cognitive difficulty (i.e., serious difficulties in concentrating, remembering, or making decisions) and three indicators of social difficulties (i.e., difficulties in making or keeping friends, bullying others, being bullied). Separate analyses were carried out, first for the number of 24-HMB guidelines met (continuous variable) and then for specific combinations (physical activity, sleep duration, screening time, screen time + sleep, screen time + physical activity, sleep + physical activity, and physical activity +screen time + sleep) of guideline recommendations (categorical variables) as independent variables in the models. Socio-demographic and medical data (age, sex, ethnicity, preterm birth status, ADHD medication, ADHD behavioral treatment, household poverty level (federal poverty level, FPL), and the highest level of education of the parents/legal guardian of the child) were included as potential confounders. For all statistical analyses, the significance level was set at p<0.05. The statistical analyses were conducted using Stata, version Stata/SE 15.1 (StataCorp LLC., College Station, TX, USA).

## Results

### Sample characteristics

This study included 3470 children and adolescents with ADHD aged 6–17 years, from the 42,777 households in the USA that provided full data. The children and adolescents had a mean age of 11.97 ± 3.48 years. The distribution of ethnicity in the sample was 69.18% white, 17.29% black or African American, and 11.01% with two or more ethnic groups, while other ethnic groups were smaller (see Table 1). The symptoms of ADHD experienced by the participants were reported as being mild (43.20%), moderate (42.29%), and severe (14.52%). Approximately a quarter of the sample was not receiving treatment at the time of the study (26.27%), a similar proportion received medication only (26.18%), approximately a third of the sample received a combination of both medication and behavioral therapy (33.15%), and a smaller group received behavioral treatment only (14.39%). The household poverty level and the education status of the adults are also presented in Table 1.

### Meeting the 24-HMB guidelines

**Fig. 1:**
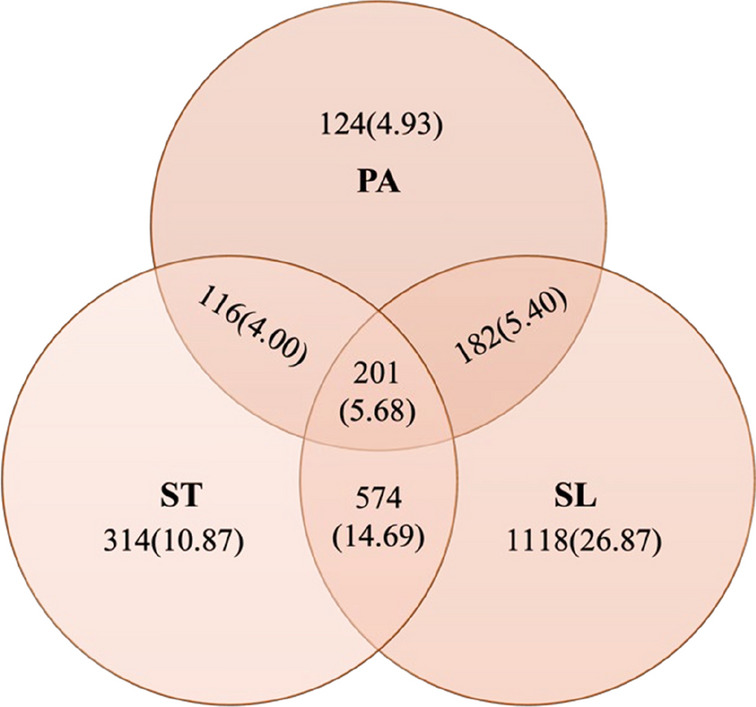
Venn diagram showing proportions of participants meeting 24-h movement guidelines. Values are n (%). N represents unweighted sample counts and % is weighted sample sizes. *PA* physical activity, *ST* screen time, *SL* sleep

Within our sample, just over a quarter (27.6%) met none of the 24-HMB guidelines, 44.8% met only one guideline, of which sleep was the most common (26.9%) and physical activity the least common (4.9%). Only 201 (5.7%) of the sample met all three 24-HMB guidelines (see Table 1 and Fig 1). The sub-groups meeting one or more of the 24-HMB guidelines are represented in the Venn diagram (Fig 1).

### Socio-demographic data and medical information

**Fig. 2:**
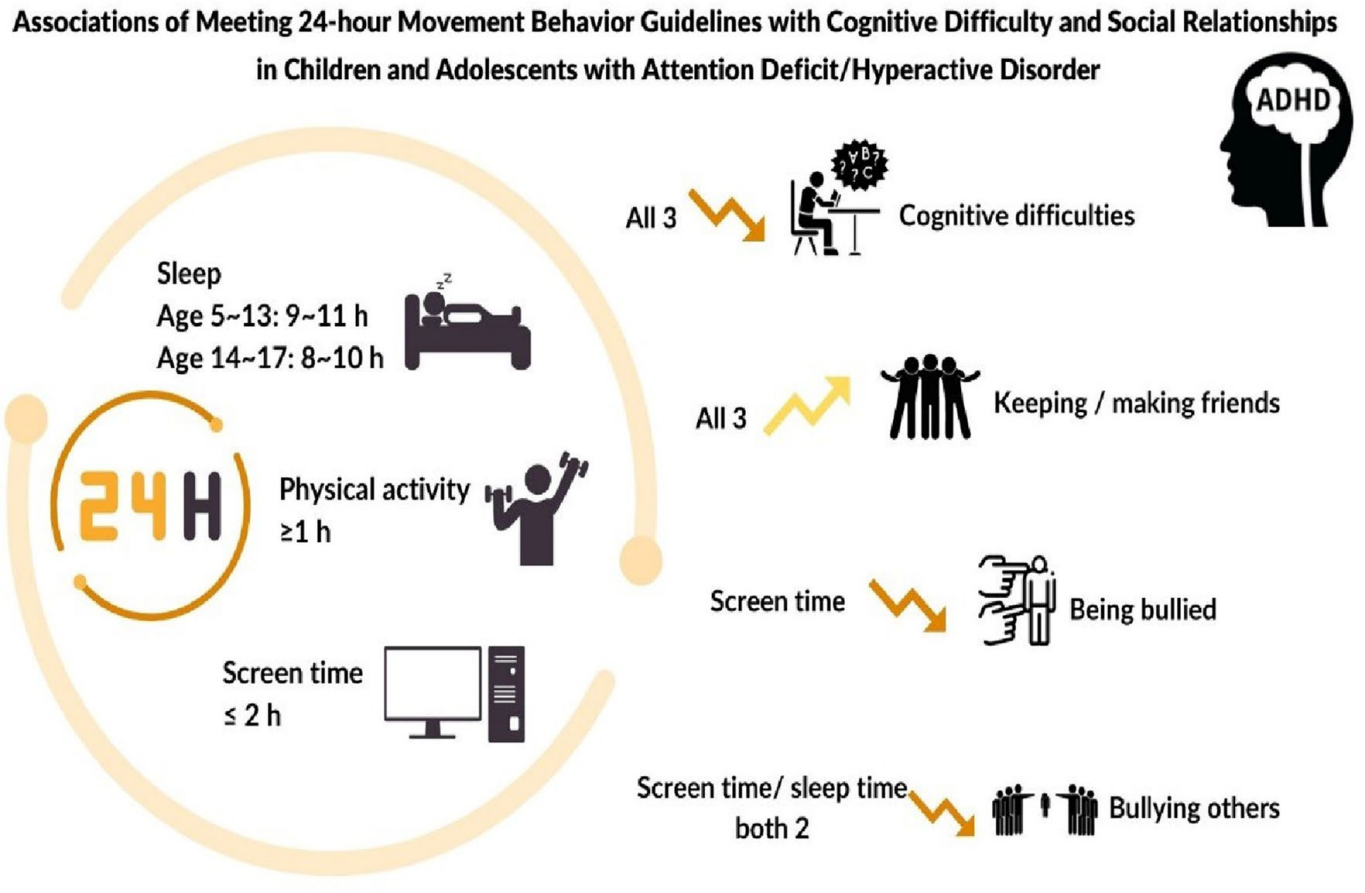
Associations of meeting 24-h movement behavior guidelines with cognitive function and social relationships among children and adolescents with ADHD

Associations between the socio-demographic data and the outcome measures were tested. When the sample was divided into two groups by age, the older group (i.e., aged 14–17 years) were significantly less bullied than the younger group (i.e., aged 6–13 years) (OR = 0.52, 95% CI 0.40–0.68, *p*<0.001). When the data was separated by sex, the odds of being bullied were significantly higher for females than for males (OR = 1.39, 95% CI 1.08–1.78, *p* = 0.01) and the odds of difficulties in concentrating, remembering, or making decisions were significantly higher for females (OR = 1.41, 95% CI 1.06–1.87, *p* = 0.02). Concerning ethnicity, the white sub-group was used as the reference group and only the native Hawaiian and other Pacific Islander group were observed to be at higher odds for difficulties in concentrating, remembering, or making decisions (OR = 9.68, 95% CI 1.37–68.41, *p* = 0.02) and higher odds of difficulties in making or keeping friends (OR = 2.88, 95% CI 1.03–8.08, *p* = 0.04) (Fig. 2).

Being overweight was associated with higher odds for difficulties in making or keeping friends (OR = 1.48, 95% CI 1.09–2.01, *p* = 0.01), but no other outcome. With regard to the severity of symptoms of ADHD, the children and adolescents with mild symptoms were used as reference group. Those with moderate symptoms had significantly higher odds of all measured outcomes related cognitive difficulties and social relationships, and those with severe symptoms were associated with the highest odds for each outcome (see Tables 2, 3, 4, 5). Concerning treatment, there was a statistically significant association found between combined behavioral treatment and medication, and higher odds of difficulties in concentrating, remembering, or making decisions (OR = 1.58, 95% CI 1.06–2.37, *p* = 0.03). Statistically significant associations also occurred between behavioral treatment only and higher odds of all three social relationship outcomes, difficulty making or keeping friends, being bullied, and bullying others (OR = 1.95, 95% CI 1.28–2.98, *p* = 0.002; OR = 1.78, 95% CI 1.20–2.65, *p* = 0.01; OR = 1.70, 95% CI 1.02–2.85, *p* = 0.04) respectively. By contrast, there were no statistically significant associations between being born 3 or more weeks early and the cognitive or social relationship outcomes. There were also no statistically significant associations between household poverty level or highest level of education of the adults and the outcomes.

**Table 2:** Associations between meeting 24-h movement behavior guidelines and outcomes of interest

Serious difficulties concentrating, remembering, or making decisions		
	Odds ratio (95% CI)	*p*
Intercept	0.55 (0.16–1.85)	0.34
Age		
6–13 years (reference)	1 (reference)	
14–17 years	0.89 (0.65–1.22)	0.48
Sex		
Male (reference)	1 (reference)	
Female	1.41 (1.06–1.87)	0.02
Overweight		
No (reference)	1 (reference)	
Yes	1.43 (0.92–2.20)	0.11
White (reference)	1 (reference)	
Black/African American	0.79 (0.47–1.30)	0.35
American Indian/ Alaska native	1.49 (0.48–4.67)	0.49
Asian	1.00 (0.47–2.14)	0.99
Native Hawaiian and other pacific islander	9.68 (1.37–68.41)	0.02
Two or more ethnicities	0.80 (0.48–1.31)	0.37
Born 3 weeks or more weeks before due date		
No (reference)	1 (reference)	
Yes	1.09 (0.76–1.55)	0.63
ADHD severity level		
Mild (reference)	1 (reference)	
Moderate	3.61 (2.69–4.85)	< 0.001
Severe	12.90 (7.39–22.50)	< 0.001
ADHD medication & behavioral treatment		
Neither behavioral treatment nor medication (reference)	1 (reference)	
Behavioral treatment and medication	1.58 (1.06–2.37)	0.03
Behavioral treatment only	1.50 (0.96–2.36)	0.08
Medication only	0.91 (0.63–1.32)	0.64
Household poverty level (		
0–99% FPL (reference)	1 (reference)	
100–400% FPL	0.79(0.50–1.25)	0.32
Highest level of education among reported adults		
Less than high school (reference)	1 (reference)	
High school (vocational/trade/business school)	1.02 (0.36–2.88)	0.97
Some college or associate degree	1.19 (0.44–3.21)	0.73
College degree or higher	1.10 (0.42–2.88)	0.85
24-HMB guidelines met (categorical)		
None (reference)	1 (reference)	
Screen time only	0.84 (0.50–1.42)	0.51
Sleep only	0.99 (0.66–1.47)	0.94
Physical activity only	1.27 (0.48–3.28)	0.64
Screen time + Sleep	0.67 (0.43–1.03)	0.07
Screen time + Physical activity	0.26 (0.12–0.53)	< 0.001
Sleep + Physical activity	0.82 (0.42–1.60)	0.56
All three	0.43 (0.24–0.78)	0.01
Prob > F	< 0.001	

*FPL* Federal Poverty Level

**Table 3:** Associations between all covariates, meeting 24-h movement guidelines and difficulties in making or keeping friends

Difficulties in making or keeping friends		
	Odds ratio (95% CI)	*p*
Age		
6–13 years (reference)	1 (reference)	
14–17 years	1.21 (0.92–1.59)	0.17
Sex		
Male (reference)	1 (reference)	
Female	1.12 (0.86–1.45)	0.42
Overweight		
No (reference)	1 (reference)	
Yes	1.48 (1.09–2.01)	0.01
White (reference)	1 (reference)	
Black/African American	0.73 (0.48–1.09)	0.13
American Indian/ Alaska native	0.84 (0.16–4.22)	0.83
Asian	1.19 (0.46–3.11)	0.72
Native Hawaiian and other pacific islander	2.88 (1.03–8.08)	0.04
Two or more ethnicities	0.90 (0.56–1.44)	0.65
Born 3 weeks or more weeks before due date		
No (reference)	1 (reference)	
Yes	1.40 (0.97–2.02)	0.07
ADHD severity level		
Mild (reference)	1 (reference)	
Moderate	2.62 (2.01–3.42)	< 0.001
Severe	8.13 (4.96–13.31)	< 0.001
ADHD medication & behavioral treatment		
Neither behavioral treatment nor medication (reference)	1 (reference)	
Behavioral treatment and medication	1.38 (0.97–1.95)	0.08
Behavioral treatment only	1.95 (1.28–2.98)	0.002
Medication only	0.80 (0.58–1.11)	0.18
Household poverty level		
0–99% FPL (reference)	1 (reference)	
100–400% FPL	1.08 (0.74–1.58)	0.70
Highest level of education among reported adults		
Less than high school (reference)	1 (reference)	
High school (vocational/trade/business school)	1.09 (0.52–2.29)	0.82
Some college or associate degree	1.48 (0.73–3.00)	0.28
College degree or higher	1.13 (0.56–2.29)	0.73
24-HMB guidelines met (categorical)		
None (reference)	1 (reference)	
Screen time only	0.96 (0.59–1.55)	0.86
Sleep only	1.25 (0.90–1.76)	0.19
Physical activity only	1.24 (0.55–2.82)	0.60
Screen time + Sleep	1.23 (0.80–1.88)	0.35
Screen time + Physical activity	0.59 (0.31–1.14)	0.11
Sleep + Physical activity	0.78 (0.44–1.40)	0.41
All three	0.46 (0.21–0.97)	0.04
Prob > F	< 0.001	

*FPL* Federal Poverty Level

**Table 4:** Associations between all covariates, meeting 24-h movement guidelines and bullying others

*Bullying others*		
	Odds ratio (95% CI)	*p*
Age		
6–13 years (reference)	1 (reference)	
14–17 years	0.74 (0.51–1.09)	0.13
Sex		
Male (reference)	1 (reference)	
Female	1.01 (0.76–1.36)	0.93
Overweight		
No (reference)	1 (reference)	
Yes	1.19 (0.79–1.36)	0.40
Ethnicity		
White (reference)	1 (reference)	
Black/African American	1.13 (0.66–1.96)	0.65
American Indian/Alaska native	2.01 (0.81–5.01)	0.14
Asian	1.04 (0.49–2.21)	0.92
Native Hawaiian and other pacific islander	3.94 (0.28–55.57)	0.31
Two or more ethnicities	0.72 (0.46–1.13)	0.16
Born 3 weeks or more weeks before due date		
No (reference)	1 (reference)	
Yes	1.14 (0.74–1.75)	0.56
ADHD severity level		
Mild (reference)	1 (reference)	
Moderate	1.69 (1.02–2.37)	0.002
Severe	3.00 (1.78–5.04)	< 0.001
ADHD medication & behavioral treatment		
Neither behavioral treatment nor medication (reference)	1 (reference)	
Behavioral treatment and medication	1.26 (0.81–1.95)	0.30
Behavioral treatment only	1.70 (1.02–2.85)	0.04
Medication only	0.66 (0.44–0.99)	0.05
Household poverty level		
0–99% FPL (reference)	1 (reference)	
100–400% FPL	0.85(0.51–1.43)	0.54
Highest level of education among reported adults		
Less than high school (reference)	1 (reference)	
High school (vocational/trade/business school)	0.76 (0.28–2.01)	0.58
Some college or associate degree	1.19 (0.48–2.96)	0.71
College degree or higher	0.80 (0.31–2.04)	0.64
24-HMB guidelines met (categorical)		
None (reference)	1 (reference)	
Screen time only	0.44 (0.26–0.76)	0.003
Sleep only	0.65 (0.44–0.95)	0.03
Physical activity only	1.05 (0.52–2.10)	0.89
Screen time + Sleep	0.60 (0.39–0.93)	0.02
Screen time + Physical activity	0.91 (0.44–1.89)	0.80
Sleep + Physical activity	0.94 (0.55–1.59)	0.82
All three	1.16 (0.45–3.01)	0.75
Prob > F	< 0.001	

*FPL* Federal Poverty Level

**Table 5:** Associations between all covariates, meeting 24-h movement guidelines and being bullied

Being bullied		
	Odds ratio (95% CI)	*p*
Age		
6–13 years (reference)	1 (reference)	
14–17 years	0.52 (0.40–0.68)	< 0.001
Sex		
Male (reference)	1 (reference)	
Female	1.39 (1.08–1.78)	0.01
Overweight		
No (reference)	1 (reference)	
Yes	1.45 (0.99–2.10)	0.05
Ethnicity		
White (reference)	1 (reference)	
Black/African American	0.74 (0.49–1.13)	0.17
American Indian/Alaska native	1.31 (0.35–4.95)	0.69
Asian	0.67 (0.31–1.44)	0.30
Native Hawaiian and other pacific islander	1.38 (0.12–16.41)	0.80
Two or more ethnicities	0.92 (0.58–1.46)	0.73
Born 3 weeks or more weeks before due date		
No (reference)	1 (reference)	
Yes	1.29 (0.92–1.81)	0.14
ADHD severity level		
Mild (reference)	1 (reference)	
Moderate	1.92 (1.49–2.49)	< 0.001
Severe	5.13 (3.12–8.41)	< 0.001
ADHD medication & behavioral treatment		
Neither behavioral treatment nor medication (reference)	1 (reference)	
Behavioral treatment and medication	1.12 (0.79–1.58)	0.53
Behavioral treatment only	1.78 (1.20–2.65)	0.01
Medication only	0.80 (0.58–1.10)	0.17
Household poverty level		
0–99% FPL (reference)	1 (reference)	
100–400% FPL	0.81 (0.54–1.21)	0.31
Highest level of education among reported adults		
Less than high school (reference)	1 (reference)	
High school (vocational/trade/business school)	0.93 (0.45–1.93)	0.84
Some college or associate degree	1.67 (0.82–3.42)	0.16
College degree or higher	1.14 (0.56–2.32)	0.72
24-HMB guidelines met (categorical)		
None (reference)	1 (reference)	
Screen time only	0.61 (0.39–0.97)	0.04
Sleep only	1.33 (0.94–1.88)	0.11
Physical activity only	2.47 (1.12–5.51)	0.03
Screen time + Sleep	0.89 (0.60–1.33)	0.57
Screen time + Physical activity	0.65 (0.35–1.19)	0.16
Sleep + Physical activity	0.88 (0.51–1.51)	0.64
All three	0.78 (0.40–1.52)	0.47
Prob > F	< 0.001	

*FPL* Federal Poverty Level

### Associations between meeting the 24-HMB guidelines and outcomes

#### Cognitive difficulties

##### Social difficulties

Concerning difficulties in making or keeping friends (Table 3), when meeting specific combinations of the 24-HMB guidelines were compared with meeting none of the 24-HMB guidelines, only those children and adolescents who met all three guidelines had significantly lower odds of difficulties in making or keeping friends (OR = 0.46, 95% CI 0.21–0.97, *p* = 0.04). In other words, the social relationships were stronger in those children and adolescents who meet all three 24-HMB guidelines than in peers who met less or none of the 24-HMB guidelines.

Children and adolescents who only met the 24-HMB guideline for screen time were found to be at lower odds of being bullied (OR = 0.61, 95% CI 0.39–0.97, *p* = 0.04). In contrast, there was a significant association between meeting the guideline for physical activity only and increased odds of being bullied (OR = 2.47, 95% CI 1.12–5.51, *p* = 0.03), as shown in Table 4. Regarding bullying others (Table 5), when meeting specific combinations of 24-HMB guidelines were compared with meeting none of the guidelines, screen time only, sleep only, and the combination of screen time and sleep were all associated with significantly lower odds of bullying others ([OR = 0.44, 95% CI 0.26–0.76, *p* = 0.003], [OR = 0.65, 95% CI 0.44–0.95, *p* = 0.03], [OR = 0.60, 95% CI 0.39–0.93, *p* = 0.02], respectively).

## Discussion

### Main findings

This cross-sectional study examined, for the first time, the associations between meeting 24-HMB guidelines by children and adolescents with ADHD aged 6-17 years and four outcome measures relating to cognitive and social difficulties in a large sample of data from the U.S. 2020 NSCH. We showed positive associations between meeting all or specific 24-HMB guidelines and a reduced risk for cognitive and social difficulties. Together, these findings suggest that meeting 24-HMB recommendations may reduce the development of cognitive and social difficulties in children and adolescents with ADHD.

While almost half of the children and adolescents with ADHD met at least one of the 24-HMB guidelines (44.8%), only a small proportion of them met all three guidelines (5.7%). Despite these data being collected during the COVID-19 pandemic, our results show a comparable pattern to those being observed during the previous NSCH 2018 cycle of the survey, in which 46.8% of children and adolescents with ADHD met at least one 24-HMB guideline and only 6.5% met all three [47]. In this context, it should also be noted that children and adolescents with ADHD are less likely to meet the 24-HMB guidelines than neurotypical children and adolescents in the same age range in which 91.2% met at least one 24-HMB guideline and 8.8% met all three [46]. Given the the evidence for the health benefits of meeting these 24-HMB guidelines, the latter findings stress the need to support children with ADHD and their caregivers to foster their ability to effectively adopt a healthy lifestyle.

### Cognitive difficulties

In our study, the analysis of meeting 24-HMB guidelines as a continuous variable showed that as the number of guidelines met increased, there were significantly lower odds for difficulties in concentrating, remembering, or making decisions. On examination of meeting specific combinations of 24-HMB guidelines, the combination of screen time and physical activity was the strongest predictor of reduced difficulties in concentrating, remembering, or making decisions. Meeting all three guidelines was also significantly associated with these measures of cognitive difficulty, but the reduction in the odds was not as pronounced as that for meeting the guidelines for screen time and physical activity combined.

Our results for physical activity and cognitive difficulties broadly agree with prior research that provided evidence for positive effects of physical activity on cognitive function in children and adolescents with ADHD [53–55]. However, previous research included many different types of physical activities and duration of physical activity interventions. For example, Benzing, Chang, and Schmidt, (2018) investigated the effects of acute sessions of 15 min of exergaming on 8–12-year- old children with ADHD and reported a post-exercise improvement of inhibition and switching performance [56]. Suarez-Manzano et al. (2018) reviewed studies that investigated the effects of physical activity on cognitive performance in children and adolescents with ADHD and concluded that physical activity for a minimum of 30 min, at a minimum intensity of 40% heart rate reserve, undertaken on a minimum of 3 days per week and a minimum of 5 weeks duration improved attention, inhibition, behavior, emotional and motor control [23]. Another systematic literature review examining the effect of physical activity on executive functions including attention, inhibition, task shifting, and working memory in children and adolescents up to age 18 years with ADHD reported the positive effects of habitual physical activity on all executive functions, but only shifting and working memory were statistically significant [57]. In addition, the authors noted that the positive effects on executive function were greater for physical activities that have a lower cognitive load compared to more cognitively demanding physical activities [57]. In line with the findings of the above-mentioned studies and systematic reviews reporting a positive influence of physical activity on cognitive performance, our study provides support for the practical application of the 24-HMB guideline for physical activity for reducing cognitive difficulties in children and adolescents with ADHD.

Concerning screen time, a review of 91 studies showed significant associations between longer screen time and higher scores for symptomatology associated with ADHD in children and adolescents [58]. For instance, Suchert et al. (2017) examined the specific activities in sedentary behavior of adolescents aged 13–17 years and found that screen time was associated with symptomatology associated with ADHD, while this was not observed for non-screen sedentary activities [58]. The lack of association with non-sedentary behavior might suggest screen time has effects beyond simple sedentary behavior, possibly due to the lack of short physical activity breaks typically observed in non-recreational sedentary behavior [59], or alternatively that recall of time spent on screen time activity is better than recall of general sedentary behavior [60]. Another possible explanation for the effect of screen time arises from evidence that blue light exposure can delay or disturb sleep [61]. In relation to this, Lissak (2018) reported that an intervention-related reduction of screen time improved ADHD-related behavior and sleep duration in children and adolescents [62]. Studies examining cognitive function in children and adults in the general population have also reported changes in the structure of the brain areas responsible for cognitive control and emotional regulation in association with addictive screen time behavior [63, 64]. Moreover, extended screen time is associated with differences in executive control performance, which, in turn, can increase distractibility [65]. Lissak (2018) reported a case- study in which the intervention included reducing screen time and the results showed reduced symptoms of ADHD behavior and improved sleep duration in the youth who also engaged successfully with school work [62]. Taken together, these findings might explain the current results showing that meeting all three 24-HMB guidelines, including sleep, was associated with reduced cognitive difficulties.

With regard to meeting the 24-HMB guideline for sleep duration alone, the association between sleep duration and measures of cognitive difficulties did not reach statistical significance in our study. This is perhaps related to bias arising from the parental self-reports. This assumption is supported by the finding that objective measures of sleep duration using accelerometers showed the mean parental estimate is up to 50.5 minutes less than the objective results [66]. Another study using actigraphy showed improved rate of cognitive processing when the sleep period for adolescents with ADHD was extended to 9.5 h compared to 6.5 h [67]. Thus, in future longitudinal studies examining recommendations for sleep duration should utilize objective measures of sleep (e.g., derived by accelerometers) rather than solely relying on subjective measures (e.g. parental reports).

#### Serious difficulties in concentrating, remembering, or making decisions

The associations between meeting the 24-HMB guidelines and the measure of cognitive difficulties (serious difficulties in concentrating, remembering or making decisions), are presented in Table 2. Our multivariable regression analysis revealed that the number of guidelines met was associated with significantly lower odds for difficulties concentrating, remembering, or making decisions (OR = 0.76, 95% CI 0.64–0.91, *p* = 0.002). When specific combinations of the 24-HMB guidelines were compared with meeting none of the guidelines, meeting a combination of both the sleep and the physical activity guidelines, or all three guidelines were associated with significantly lower odds of suffering from difficulties in these cognitive abilities (OR = 0.26, 95% CI 0.12–0.53, *p *< 0.001 and OR = 0.43, 95% CI 0.24–0.78, *p* = 0.01, respectively).

#### Social difficulties

##### Making and keeping friends

In the current study, children and adolescents who met all three 24-HMB guidelines had significantly lower odds of difficulties in making or keeping friends, reflecting better social relationships with peers. Well-developed social skills are important for success in academic [68] and work environments as well as social relationships for all children and adolescents including those with developmental challenges [69]. Children and adolescents with ADHD whose symptoms may include intrusive, impulsive, or aggressive behavior, can experience barriers to successful social interactions [60]. Such social relationship difficulties can lead to reduced self-esteem and poor mental health, including depression [70]. The latter is supported by a large study that examined longitudinal data from 2950 people who had been diagnosed with ADHD by the age of 7.5 years and observed that symptoms in childhood were associated with an increased risk of depression at age 17.5 years [71]. Furthermore, this increased risk of depression was mediated by both social relationships with peers and academic achievement at 16 years of age [71]. Considering our findings in the context of the previous literature, it seems reasonable to suggest that those who meet the 24-HMB guidelines are more likely to have better social relationships and might also have a lower risk of depression and thus a better chance of academic achievement. However, future longitudinal research is needed to empirically test this hypothesis.

#### Social difficulties

##### Being bullied

Children and adolescents in our study who met the 24-HMB guideline for screen time only were found to be at lower odds of being bullied. This finding may indicate that those who are less dependent on screen-based activities are also less vulnerable to being bullied. Previous research on adolescents with ADHD indicated that a high dependence on screen-based recreational activities is strongly associated with low self-esteem [72]. Speculatively, a lower self-esteem might make them more vulnerable to being bullied. In contrast, our results revealed an association between meeting the 24-HMB guidelines for physical activity and increased odds of being bullied. A possible explanation might be that the experience of being bullied increased the motivation to engage in physical activity, possibly to increase self-esteem [73, 74]. Bejerot et al. (2022) who examined possible associations between ADHD and bullying behaviors in a cross-sectional study, found that for participants who had been diagnosed with ADHD and that also suffer from poor motor skills (i.e, ball dexterity, coordination, or agility performance), have a higher risk to being bullied [75]. Therefore, another possible explanation for the increased odds of meeting the physical activity guidelines might be that these young people sought to improve physical activity skills to prevent the bullying. Longitudinal studies are needed to examine these theoretical assumptions.

#### Social difficulties

##### Bullying others

Our results showed that meeting the 24-HMB guideline for sleep only, screen time only, and the combination of screen time and sleep were all associated with significantly lower odds of bullying others. Improved sleep has been associated with reduced antisocial behavior in school [62]. Li et al. (2021) examined NSCH data from 2011 to 12 for adolescents and found that meeting the age-appropriate sleep target mediated the association between increased MVPA and less bullying behavior [76]. Further, Moreau et al. (2013) found that executive functioning was positively associated with sleep duration in children with ADHD [77]. Previously, Unnever and Cornell (2003) had found that those with ADHD taking medication were more likely to bully others, which is perhaps related to a poorer self-control [78]. Taken together, the evidence presented above suggests that a longer sleep duration contributes to reduced bullying behavior, which in turn might be related to a sleep-related increase of inhibition performance.

With regard to our results for meeting the 24-HMB guideline for screen time associated with lower odds of bullying others, previous research might provide an explanation for the current findings. Yen et al. (2014) found that addictive screen time behavior was associated with decreased social coping in adolescents aged 11 to 18 years old with a diagnosis of ADHD [72]. In addition, there is some evidence to suggest increased use of electronic devices, particularly for rapid response gaming may stimulate increased hyper vigilance and stress response, and increase ADHD symptoms [62]. There is also evidence from a study that investigated the frequency of digital media use in adolescents over 2 years and revealed higher frequency of digital media use was associated with higher level of ADHD symptoms [79]. While the results from our cross-sectional study do not indicate a direction to the association between meeting the 24-HMB guideline for screen time and reduced risk of bullying others, the literature suggests limiting the screen time may support social coping, and/or reduce exposure to stimulation that may cause hyper vigilance, stress or increased ADHD symptoms [28].

### Implications and practical applications

In conjunction with findings of previous research [24, 25, 48], the results of our study suggest that meeting all three of the 24-HMB guidelines is associated with reduced cognitive and social difficulties in children and adolescents with ADHD. Accordingly, our findings support the promotion of the 24-HMB guidelines for children and adolescents with ADHD and their caregivers.

A key finding of our study is that meeting the 24-HMB guideline for non-educational (recreational) screen time made a substantial contribution to reduced odds for negative results for all four outcomes relating to cognitive and social difficulties, indicating the children and adolescents are very attracted to using electronic devices for recreation including games [62, 80, 81]. Therefore, it seems reasonable to speculate that some elements that attract them to use the virtual environment might be useful to stimulate learning and specific movement behaviors (e.g. engagement in MVPA). For example, promotion of physical activity through exergaming could be a valuable intervention strategy to reach this cohort [82], while meeting 24-HMB guidelines for non-educational screen time [28, 29].

## Strengths and limitations

A strength of this study is the sample of 3740 sets of data on children and adolescents derived from the 42,777 households who provided full responses to the nationwide collection of the NSCH 2020 survey. However, a disadvantage of the current study is the cross-sectional design which does not provide information on possible causal relationships between variables and thus necessitates further research using longitudinal studies to examine the causal mechanisms supporting our observations. Furthermore, as the current findings are based on information provided by the parent or guardian of the child/ adolescent, our results may be prone to reporting biases. The latter point is particularly applicable to sleep duration which is typically over estimated by the parents, especially for children with poor sleep efficiency [66].

While the measures for cognitive and social difficulties included in the NSCH survey provide some relevant data for the outcomes of interest, other validated measures for cognitive difficulties [83] and social difficulties [84] could be used in future research including controlled studies designed to examine the effects of meeting 24-HMB guidelines on these outcomes in children and adolescents with ADHD. Future studies should be designed to use objective and reliable measures of movement behaviors in order to gain a more nuanced understanding of their influence on health-related outcomes. For example, using objective measures for sleep timing, sleep quality and sleep duration [85, 86], could increase the robustness of the observations and lead to a more fine-graded understanding of the effects of specific movement behaviors. Likewise, prospective controlled research is needed to examine whether the time of day, days of the week, or specific type of physical activities undertaken by the children effect the cognitive or social difficulties outcomes in children with ADHD.

Poitras et al. (2016), who undertook a systematic review, observed that children and adolescents in the general population could benefit from the recommended amount of MVPA (i.e. 60 minutes per day), even if it was accumulated in small bouts over the day [87]. However, Schmidt et al. (2015), showed that while both team games and aerobic exercise in children aged 10–12 years improved measures of aerobic fitness, only the team games improved executive function [88].Thus, the dose-response relationship considering qualitative (i.e. type of physical activity) and quantitative characteristics of movement behaviors (i.e. duration of physical bouts) should be examined in more detail in future studies. In addition, it would be an interesting topic for future research to compare the associations between the 24-HMB guidelines and the same outcome measures for cognitive and social difficulties between the current cohort of children with ADHD and a matched sample of the same survey population without a diagnosis of ADHD.

## Conclusion

This cross-sectional study examined whether meeting 24-HMB guidelines—including recommendations concerning physical activity, sedentary behavior, and sleep—is, in a large sample of US children and adolescents with ADHD, associated with specific social and cognitive outcomes. The results of the current study revealed that meeting all three 24-HMB guidelines was associated with reduced odds of the occurrence of one or more negative outcomes for cognitive and social difficulties. Screen time, as a measure of sedentary behavior, was associated with all cognitive and social outcomes of interest, including serious difficulty concentrating, remembering, or making decisions; difficulty in making friends or keeping friends; being bullied; or bullying others. Furthermore, meeting the 24-HMB guideline for physical activity is linked to less cognitive difficulty and less social difficulty (i.e. making and keeping friends). Meeting the sleep recommendation of the 24-HMB guidelines is associated with less social difficulties—namely bullying others. The results of this study together with the previous literature on the benefits of adhering to the 24-HMB guidelines suggest the need to support children with ADHD and their caregivers to foster their ability to effectively adopt a healthy lifestyle. While further research is required to determine causal pathways, our results highlight the relevance of the 24-HMB guidelines as part of healthy lifestyle behaviors to enhance cognitive and social function for children and adolescents with ADHD.

## Data Availability

The data for this study were selected from publicly available information provided by the United States Census Bureau repository, https://www.census.gov/programs-surveys/nsch/data/datasets.html.

## Abbreviations

24-HMB: 24-hour movement behaviors
ADHD: Attention deficit/hyperactive disorder
FPL: Federal poverty level
PA: Physical activity
SB: Sedentary behaviors
SL: Sleep duration
